# Prognostic Significance of Growth Pattern in Predicting Outcome of *Opisthorchis viverrini*-Associated Distal Cholangiocarcinoma in Thailand

**DOI:** 10.3389/fpubh.2022.816028

**Published:** 2022-05-16

**Authors:** Waritta Kunprom, Chaiwat Aphivatanasiri, Prakasit Sa-ngiamwibool, Sakkarn Sangkhamanon, Piyapharom Intarawichian, Walailak Bamrungkit, Malinee Thanee, Piya Prajumwongs, Watcharin Loilome, Narong Khuntikeo, Attapol Titapun, Apiwat Jareanrat, Vasin Thanasukarn, Tharatip Srisuk, Vor Luvira, Kulyada Eurboonyanun, Julaluck Promsorn, Supinda Koonmee

**Affiliations:** ^1^Cholangiocarcinoma Screening and Care Program (CASCAP), Khon Kaen University, Khon Kaen, Thailand; ^2^Cholangiocarcinoma Research Institute, Khon Kaen University, Khon Kaen, Thailand; ^3^Department of Pathology, Faculty of Medicine, Khon Kaen University, Khon Kaen, Thailand; ^4^Department of Biochemistry, Faculty of Medicine, Khon Kaen University, Khon Kaen, Thailand; ^5^Department of Surgery, Faculty of Medicine, Khon Kaen University, Khon Kaen, Thailand; ^6^Department of Radiology, Faculty of Medicine, Khon Kaen University, Khon Kaen, Thailand

**Keywords:** distal cholangiocarcinoma, 8th AJCC staging system, growth pattern, classification, prognosis

## Abstract

Distal cholangiocarcinoma (dCCA) is a rare type of CCA in Asia, even in *Opisthorchis viverrini*-prevalent Northeastern Thailand. The clinical ambiguity and imprecision of diagnosis surrounding this malignancy result in high mortality due often to advanced/metastatic disease on presentation. We aim to identify a prognostic factor that can improve the performance stratification and influence the outcome of dCCA patients after curative resection. A total of 79 patients who underwent curative-intended surgery for dCCA was enrolled. Possible risk factors for survival were analyzed with log-rank test, and independent factors with Cox regression model. dCCA patients were staged and classified according to the 8th edition the American Joint Committee on Cancer (AJCC) Staging Manual. Results were then compared with the revised classification employing the prognostic factor identified from multivariate analysis. Multivariate analysis revealed that growth pattern (*p* < 0.01) and distant metastasis (*p* = 0.012) were independent factors. Growth patterns comprise intraductal (ID), periductal infiltrating (PI), mass-forming (MF), and mixed types. When dCCA patients were grouped into those having good and poor outcomes (with and without ID components, respectively). The survival outcomes significantly differed among patients with and without ID components, which was better than with the 8th AJCC staging system in our cohort. Furthermore, Chi-square test showed that patterns without ID components (PI, MF, PI + MF) correlated with lymph node and distant metastasis. Therefore, classification of dCCA patients after curative-intended surgical resection based on growth pattern provides additional beneficial information for the prediction of survival in dCCA patients.

## Introduction

Cholangiocarcinoma (CCA) is cancer that originates in the intra- or extrahepatic biliary tree (1). CCA is rare in most countries, with an incidence of <6 per 100,000 population. However, the incidence is exceptionally high in Chile, Bolivia, Korea, and Thailand. The Northeastern (NE) region of Thailand has the highest incidence worldwide (85 per 100,000 population per year) (2–4). Studies have substantially demonstrated that the liver fluke, *Opisthorchis viverrini* (OV) is associated with the high incidence of CCA patients in Thailand (5–10). OV is welldocumented with reports of several possible carcinogenic mechanisms (4, 11, 12). CCA has high mortality rates because of difficulties encountered in early diagnosis with patients often presenting with metastatic disease (13). Accurate stratification and staging of CCA patients can potentially guide patient counseling, prognosis prediction, and treatment options.

The classification of CCA is based on anatomical localization. Although intrahepatic (iCCA), perihilar (pCCA) and distal CCA (dCCA) have similarities, there are some significant inter-and intra-tumoral dif-ferences that can influence the pathogenesis and outcome (14). This study focuses on dCCA because of the dismal outcome and the lack of capability of current staging systems to accurately classify and stratify patients after curative-intended surgery for optimum management (15, 16).

According to The American Joint Committee on Cancer (AJCC) and The Union for International Cancer Control (UICC) definition, dCCA arises from the extrahepatic biliary tree from the confluence of the common hepatic bile duct with the cystic duct to form the common bile duct and all the way through the pancreatic portion to the ampulla of Vater (17). It has been reported to occur relatively more frequently in Western countries and North America, accounting for approximately 30% of all CCA (18–20). The incidence is low in Southeast Asia, accounting for approximately 10% of all CCA in Thailand (15, 16). Almost all dCCA patients have poor outcomes due to advanced disease at presentation with lymph node and distant metastasis (21). Surgery is usually the first choice for palliative treatment, while chemotherapy and radiotherapy are secondary options (22). The 5-year survival time and rate are approximately 20 months and 25%, respectively (16, 18–20, 23). In addition, alternative therapy such as immunotherapy with immune checkpoint inhibitors (ICI), has been suggested by Rizzo A. and the team for advanced CCA treatment. They reported that ICI provided effective treatment in phase I-III of advanced CCA (24, 25). Although ICI is deemed as new strategy to combat advanced CCA, there remains room for investigations to confirm performance and safety. There is, therefore, a need to improve the clustering of dCCA patients for precise prognostication and management.

The AJCC/UICC system determines the classification and staging of cancers worldwide. The 8th edition of AJCC/UICC Staging Manual was recently updated from the 7th edition with improved classification ability. A validation study performed by Jun et al. on 200 surgically resected dCCA patients showed that the new T [T1-T3 using depth of invasion (DOI)] and *n* (N1 and N2 depending on the number of lymph node metastases) categories afforded better separation of each stage and, hence, more accurate prognostic prediction than the 7th edition (26, 27). Kang et al. compared the classifying ability of the two editions in 293 patients who had curative-intended surgery. The 7th edition showed low-performance separation of T1 vs. T2 and T2 vs. T3, and no significant difference in the 5-year survival rate, whereas the 8th edition provided a significant difference in separation of T1 vs. T2 and T2 vs. T3, leading to better prognostic predictability (28). As far as inclusion of DOI and number of lymph node metastasis is concerned, the verdict is still out (29–32). There has, however, been increasing studies proposing revisions of the updated 8th edition and alternative staging systems for classification (33–37).

This study aims to validate the 8th AJCC staging system and propose additional prognostic factors suitable for dCCA patients in our cohort.

## Methods

### Patients

Between 2002 and 2017, 84 patients were diagnosed with dCCA at the Srinagarind Hospital, Faculty of Medicine, Khon Kaen University, Thailand. Patients with small biopsies and those who survived <30 days after surgery with probable perioperative causes of death were excluded. A total of 79 patients with curative-intended surgery was finally included. The follow-up time was at least 5 years. This study was approved by the Ethics Committee for Human Research, Khon Kaen University (HE641499).

### Recorded Data

Intraoperative data collection included sex, age, sample size, tumor size, growth patterns, surgical margin, and characteristics of surrounding organs. The specimens were examined with relevant tissue blocks taken by a pathologist for routine tissue processing. Formalin-fixed paraffin-embedded (FFPE) tissue blocks were sectioned at 5 microns (38) and stained with hematoxylin and eosin (H&E). The 2019 WHO classification criteria was adopted for pathological diagnosis (39). Under light microscopy, the following histomorphological data were recorded, namely, growth patterns, histological type, histological grade, surgical margin, lymphovascular invasion, and lymph node metastasis. Evidence for distant metastasis was retrieved from the medical records. Finally, the gross examination and pathological findings were correlated with the 8thAJCC Staging Manual (40).

### Growth Pattern Estimation

The resection specimens were trimmed and photographed with the tumor growth pattern/s recorded at the time of grossing followed by subsequent histological confirmation. The growth patterns comprised intraductal (ID), peridutal infiltrating (PI) and mass-forming (MF) patterns. The patterns were estimated in increments of 10% to establish the proportion of each pattern (ID, PI or MF) or combinations of patterns (ID + PI, ID + MF, PI + MF or ID + PI + MF).

### Pathological Diagnosis

There were four major histological types comprising papillary adenocarcinoma, tubular adenocarcinoma, papillotubular adenocarcinoma, and adenocarcinoma (NOS). Papillary, tubular and papillotubular adenocarcinomas were classified into well or moderately differentiated cancers (2019 WHO classification) (39). Adenocarcinoma, NOS, was defined as poorly differentiated bile duct cancer, lacking wellformed papillary or tubular structures.

### Statistical Analysis

Only patients with complete datasets were included in the statistical analyses. Statistics for categorical data were performed with the χ2-Test. The survival rate and median survival time from the date of surgery for dCCA until death from dCCA used the Kaplan-Meier model which is applicable for survival analyses; the Log-rank test was used to compare factors. Perioperative causes of death were excluded from this analysis. Multivariate analysis was performed using the Cox regression model to determine prognostic factors. For the percentage of growth pattern decision criteria, a 20% growth pattern estimation cut-off value was used as this showed significantly different overall survival (OS) between each type of growth pattern. All statistical analyses were performed using SPSS version 23. *P*-values of <0.05 were considered to be statistically significant.

## Results

### Clinicopathological Characteristics of Risk Factors on 1-, 3- and 5-Year Survival of Distal Cholangiocarcinoma Patients

This pilot study of 79 dCCA patients was analyzed by a surgeon-pathologist team. The clinicopathological features are described in Table 1. The median age was 59 years range, 3,4-79 years. Comparison of overall survival (OS) and 1-, 3- and 5-year survival rates between patients with age ≤59 (*n* = 38, 48.1%) and those >59 (*n* = 41, 51.9%) years showed no significant difference. There were 55 males (69.6%) and 24 females (30.4) with no gender differences on OS and 1-, 3- and 5-year survival rates.

**Table 1:** Overall survival, 1-, 3- and 5-year survival rates and univariate analysis of possible risk factors on survival of distal cholangiocarcinoma patients.

**Feature**	**79 (100)**	**OS (month)**	**Survival rate (years)**			**Univariate analysis HR (95%CI)**	***P***
			**1**	**3**	**5**		
Age							
≤59	38 (48.1)	17	60.5%	26.3%	7.9%	1	0.488
>59	41 (51.9)	16	65.9%	9.8%	4.9%	1.18 (0.74–1.87)	
Gender							
Male	55 (69.6)	15	58%	14.5	1.8%	1	0.667
Female	24 (30.4)	22	75%	25%	16.7%	0.61 (0.37–1.03)	
Tumor size (cm)							
≤2	26 (32.9)	16	65.4	19.2%	7.7%	1	0.869
>2	18 (22.8)	17	72.2%	16.7%	5.6%	1.06 (0.56–1.98)	
Unknown	35 (44.3)	–	–	–	–		
Surgical margin							
R0	54 (59.1)	23	70.4%	20.4%	7.4%	1	0.013
R1	25 (40.9)	11	48%	12.0%	0%	1.86 (1.14–3.04)	
Growth pattern							
ID	13 (16.5)	40	100%	61.5%	30.8%	1	
PI	25 (31.6)	5	28%	0%	0%	12.17 (5.19–28.59)	<0.001
MF	9 (11.4)	11	44.4%	0%	0%	8.39 (3.09–22.85)	<0.001
Mixed types	32 (40.5)	24	71.9%	18.8%	3.1%	2.93 (1.38–6.24)	0.005
Histological type							
Papillary adenocarcinoma	29 (36.6)	15	62.1%	24.1%	6.9%	1	
Tubular adenocarcinoma	32 (45.6)	16	65.6%	15.6%	9.4%	1.08 (0.64–1.83)	0.769
Papillotubular adenocarcinoma	9 (11.4)	25	77.8%	1.1%	0%	1.06 (0.49–2.28)	0.882
Adenocarcinoma, NOS	9 (11.4)	10	44.4%	1.1%	0	1.41 (0.66–3.02)	0.661
Histological grade							
Welldifferentiated	70 (88.6)	17	64.3%	18.6%	7.1%	1	0.291
Moderately/Poorly differentiated	9 (11.4)	16	55.6%	11.1%	0%	1.46 (0.72–2.96)	
8th AJCC staging system							
T categories							
T1	10 (12.7)	31	80%	40%	20%	1	
T2	38 (48.1)	14	55.3%	13.2%	5.3%	2.23 (1.03–4.82)	0.042
T3	29 (36.7)	17	69%	13.8%	0%	2.32 (1.05–5.14)	0.038
T4	2 (2.5)	–	–	–	–	–	–
Lymph node metastasis (N)							
N0	51 (64.6)	23	70.6%	21.6%	9.8%	1	0.049
N1	26 (32.9)	12	53.8%	11.5%	0%	1.64 (1.00–2.68)	
N2	2 (2.5)	–	–	–	–	–	–
Distal metastasis (M)							
M0	69 (87)	22	66.7%	20.3%	7.2%	1	
M1	10 (13)	3	40%	0%	0%	2.34 (1.19–4.62)	0.012

*R, Surgical margin; Growth pattern refers: ID, intraductal growth; PI, periductal infiltrating growth; MF, mass forming growth and mixed types, ID + PI, ID + MF, PI + MF and ID + PI + MF; Adenocarcinoma, NOS, Adenocarcinoma not otherwise specified; AJCC, American Joint Committee on cancer; T, the primary tumor; N, the lymph node metastasis; M, the distal metastasis; Unknown, unknown tumor size*.

The median tumor size was 2 cm (range, 0.2–11 cm). Patients were categorized into those with tumor size ≤2 cm (*n* = 26, 32.9%), >2 cm (*n* = 18, 22.8%), and of unknown size (*n* = 35, 44.3%). No significant statistical difference of OS was observed between the first two groups (*p* = 0.869).

**Figure 1:**
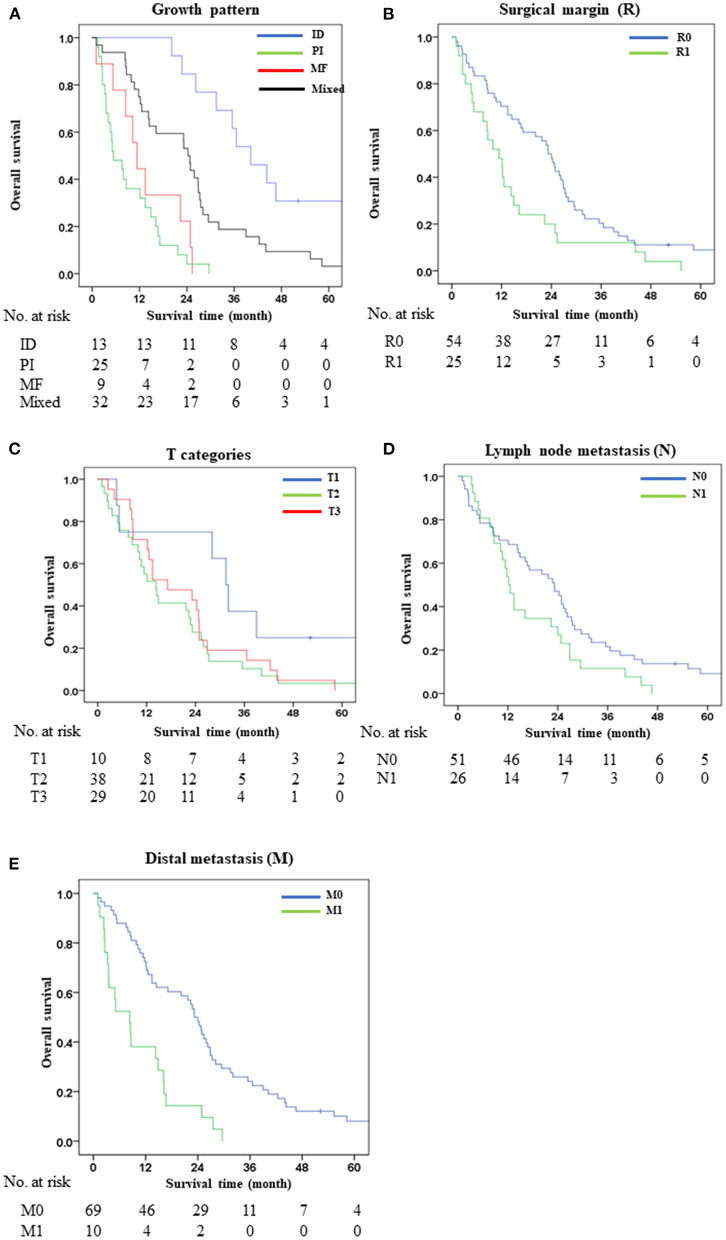
1- 3- and 5-year survival rates of possible risk factors on poor survival of distal cholangiocarcinoma patients. **(A)** Surgical margin, **(B)** Growth pattern, **(C)** T categories, **(D)** Lymph node metastasis, and **(E)** Distant metastasis.

Growth pattern was divided into four groups comprising ID (*n* = 13, 16.5%), PI (*n* = 25, 31.6%), MF (*n* = 9, 11.4), and mixed types (ID + PI, ID + MF, PI + MF and ID + PI + MF) (*n* = 32, 40.5%). ID was used as a baseline for good survival with OS of 40 months (mo.). Results showed that ID had significantly better OS than PI (OS = 40 vs. 5, HR = 12.17, *p* < 0.001), MF (OS = 40 vs. 11, HR = 8.39, *p* < 0.001), and mixed types (OS = 40 vs. 24, HR = 2.93, *p* = 0.005). Of note, the 1-, 3- and 5-year (y) survival rates of ID were higher than for PI (1y = 100 vs. 28%, 3y = 61.5 vs. 0%, and 5y = 30.8 vs. 0%), MF (1y = 100 vs. 44.4%, 3y = 61.5 vs. 0%, and 5y = 30.8 vs. 0%), and mixed types (1y = 100 vs. 71.9%, 3y = 61.5 vs. 18.8%, and 5y = 30.8 vs. 3.1%) (Figure 1A).

The surgical margin was assessed histologically. R0 (*n* = 54, 59.1%) was free from tumor and R1 (*n* = 25, 40.9%) was involved by tumor. The OS of R0 was significantly greater than R1 (OS = 23 vs. 11 mo., HR = 1.86, *p* = 0.013). Survival rates at 1-, 3- and 5-years showed R0 to be better than R1 (1y = 70.4 vs. 48%, 3y = 20.4 vs. 12%, and 5y = 7.4 vs. 0%) (Figure 1B).

Histological grade, using welldifferentiated adenocarcinoma as a reference group, showed no significant difference in OS and survival rate with moderately/poorly differentiated carcinomas (OS = 17 vs. 16 mo., HR = 1.46, *p* = 0.291). For histological types, papillary carcinoma showed no significant difference of OS and survival rate when compared with tubular carcinoma (OS = 17 vs. 16 mo., HR = 1.08, *p* = 0.769), papillotubular carcinoma (OS = 17 vs. 25 mo., HR = 1.06, *p* = 0.882), and adenocarcinoma, NOS (OS = 17 vs. 10 mo., HR = 1.41, *p* = 0.661). This study showed that histological grade and type had no apparent effect on OS and survival rate of dCCA.

TNM categories according to the 8th AJCC staging system were used. T categories comprised T1 (*n* = 10, 12.7%), T2 (*n* = 38, 48.1%), T3 (*n* = 29, 36.7%), and T4 (*n* = 2, 2.5%). Results showed that OS and survival rate of T1 were significantly better than T2 (OS = 32 vs. 14 mo., HR = 2.23, *p* = 0.042), and T3 (OS = 32 vs. 17 mo., HR = 2.32, *p* = 0.038); while T4 was not statistically calculated due to insufficient patients. For 1-, 3- and 5-year survival rates, T1 was higher than T2 and T3 in all three periods (1y = 80 vs. 55.6% and 69%; 3y = 40 vs. 13.2% and 13.8%; and 5y = 20 vs. 5.3% and 0%, respectively). Lymph node metastasis (N) comprised N0 (*n* = 51, 64.6%), N1 (*n* = 26, 32.9%), and N2 (*n* = 2, 2.5%). N1 had remarkably shorter survival time and rate than N0 (OS = 12 vs. 23 mo., HR = 1.64, *p* = 0.049). Distant metastasis (M) included M0 (*n* = 58, 72.2%) and M1 (*n* = 21, 27.8%). Survival analysis showed that M1 had significant shorter OS and survival rate than M0 (OS = 3 vs. 22 mo., HR = 2.58, *p* = 0.012; and 1-, 3- and 5-year survival rates = 40 vs. 66.7%, 0 vs. 20.3%, and 0 vs. 7.2%, respectively). Metastatic sites included liver (*n* = 3), omentum (*n* = 3), peritoneum (*n* = 3), and hepatoduodenal tissue (*n* = 1) (Figures 1C–E). Significant data in univariate analysis were further analyzed by multivariate Cox regression analysis for identifying predictive prognostic risk factors.

### Multivariate Analysis of Significant Risk Factors on Survival of Distal Cholangiocarcinoma Patients

To investigate prognostic factors, the clinical features that showed significant statistical difference in survival by univariate analysis were selected to be analyzed by multivariate Cox regression analysis. The adjustment variables included surgical margin, growth pattern, T category, lymph node metastasis, and distant metastasis. This study demonstrated that growth pattern was an independent factor for dCCA. With ID as a reference group for comparison of growth patterns in multivariate analysis, results revealed that PI, MF, and mixed types had hazard ratios markedly higher than pure ID (HR = 12.36, 6.28, and 3.11; *p* < 0.001, *p* = 0.001 and *p* = 0.007, respectively). In addition, positive surgical margin, R1, had significantly higher hazard ratio than negative surgical margin, R0 (HR = 1.86, *p* = 0.028). This finding suggested that growth patterns (PI, MF, and mixed types) and surgical margin were poor prognostic factors for dCCA patients (Table 2).

**Table 2:** Multivariate analysis of possible risk factors on survival of distal cholangiocarcinoma patients.

**Feature**	**No. of patients (79)**	**Multivariate analysis HR (95%CI)**	***P***
Surgical margin (R)			
R0	54	1	
R1	25	1.86 (1.07–3.24)	0.028
Growth pattern			
ID	13	1	
PI	25	12.36 (4.65–32.83)	<0.001
MF	9	6.28 (2.21–17.81)	0.001
Mixed types	32	3.11 (1.36–7.13)	0.007
8th AJCC			
T categories			
T1	10	1	
T2	38	1.74 (0.76–3.96)	0.190
T3	29	1.80 (0.78–4.18)	0.171
T4	2	–	–
Lymph node metastasis (N)			
N0	51	1	
N1	26	1.43 (0.84–2.43)	0.184
N2	2	–	–
Distant metastasis (M)			
M0	69	1	
M1	10	0.97 (0.41–4.21)	0.934

### Classification of Distal Cholangiocarcinoma by 8th AJCC Staging System and the 1-, 3- and 5-Year Survival of the Main Stages

**Figure 2:**
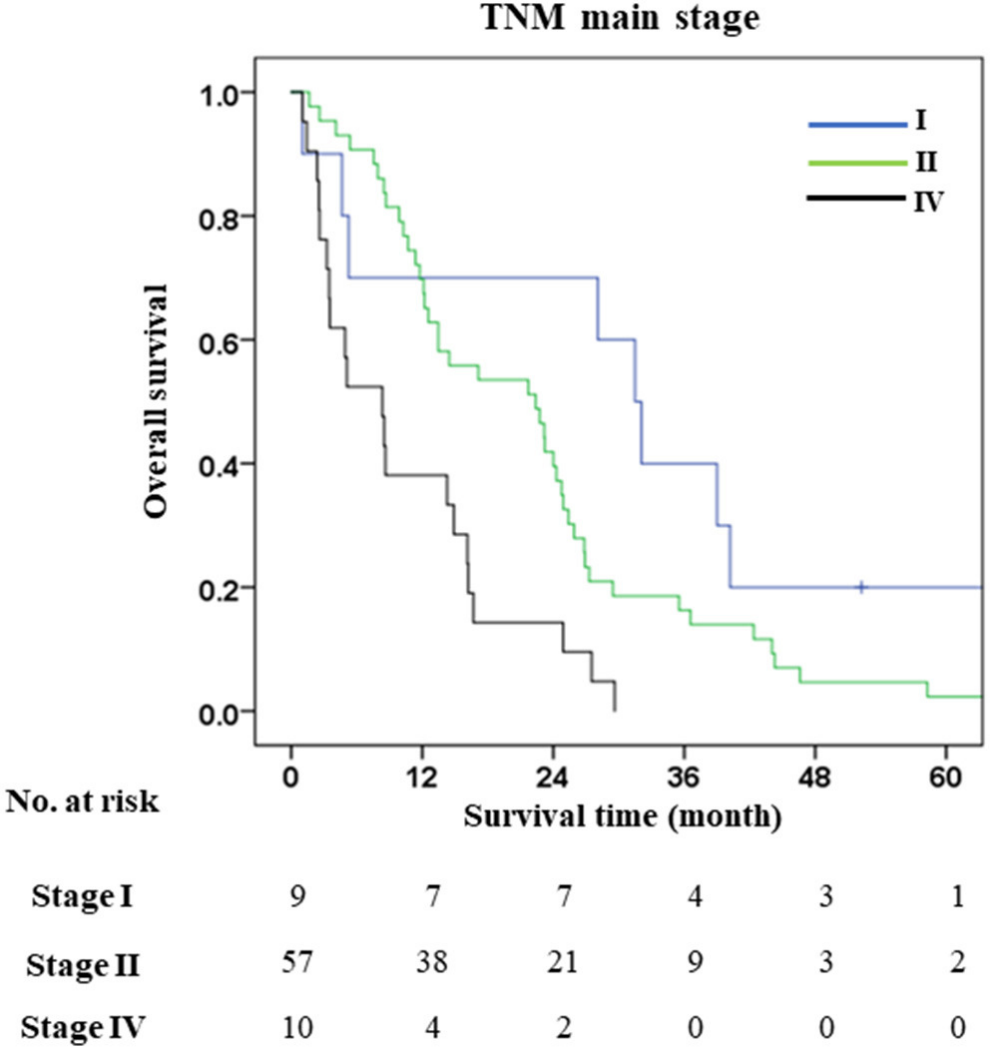
1-3- and 5-year survival rates of distal cholangiocarcinoma patient classified with 8th AJCC staging system.

Based on the 8th AJCC staging system, this study cohort was classified into stage I (*n* = 9), IIA (*n* = 23), IIB (*n* = 34), IIIA (*n* = 2), IIIB (*n* = 1), and IV (*n* = 10). Due to the small number of patients in stages IIIA and IIIB, this study analyzed only stages I, IIA, IIB, and IV to calculate survival time and rate (Table 3; Figure 2). Although OS and survival rate of stage I was better than IIA (OS = 32 vs. 22, HR = 2.04, *p* = 1.04), IIB (OS = 32 vs. 13, HR = 2.61, *p* = 0.023), and IV (OS = 32 vs. 3, HR = 4.98, *p* = 0.001), the stratification result showed unsatisfactory performance due to the lack of stages IIIA and IIIB which might affect the precision of diagnosis and prognosis impacting on the efficacy of any treatment plans. For this reason, an alternative classification was deemed more suitable for dCCA patients in Thailand.

**Table 3:** Overall survival and 1-, 3- and 5-year survival rates of distal cholangiocarcinoma patients classified with 8th AJCC staging system.

**Feature**	**N (%) 79 (100)**	**OS (month)**	**Survival rate (years)**			**Univariate analysis HR (95%CI)**	***P***
			**1**	**3**	**5**		
8th AJCC, TNM stage							
Stage I	9 (11.4)	32	77.8%	33.3%	11.1%	1	
Stage IIA	23 (29.1)	22	73.9%	17.4%	8.7%	2.04 (0.86–4.84)	0.104
Stage IIB	34 (43)	13	61.8%	14.7%	0%	2.61 (1.14–5.95)	0.023
Stage IIIA	2 (2.5)	–	–	–	–	–	–
Stage IIIB	1 (1.3)	–	–	–	–	–	–
Stage IV	10 (12.7)	3	40%	0%	0%	4.98 (1.85–13.42)	0.001

Multivariate analysis showed that growth pattern was an independent factor for predicting survival of dCCA patients. In order to study whether the presence of ID component is associated with good or poor outcome (ID vs. PI/MF patterns) and whether it influenced the outcome of the mixed types, patients were divided 5 groups—ID (13/79), PI (25/79), MF (9/79), PI + MF (9/79), and mixed types with ID component (23/79) [ID + PI (19/79), ID + MF (2/79), and ID + PI + MF (2/79)].

### Survival Analysis After Subclassification Based on Growth Patterns

**Figure 3:**
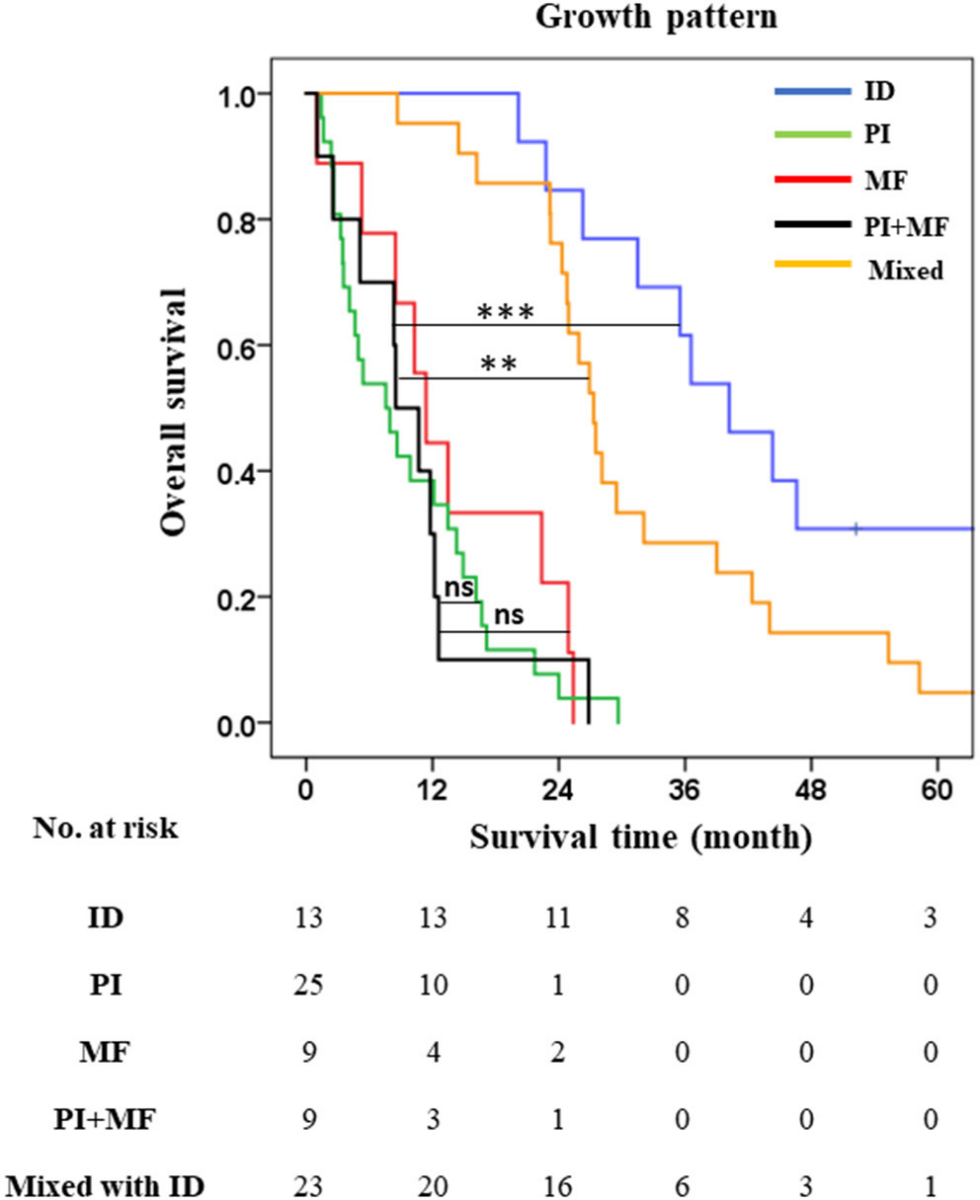
1-3- and 5-year survival rates of five subclassifications of growth pattern. **p < 0.01 and ***p < 0.001.

This study showed that growth pattern, especially PI, MF, and mixed types, was an independent factor for poor outcome in dCCA; the corollary being that presence of ID component favored good outcome. Of the four combinations in the mixed types, PI + MF lacks ID components. To investigate whether PI + MF showed shorter survival when compared with pure ID and mixed types with ID component, the growth pattern was divided into five groups comprising ID (13/79), PI (25/79), MF (9/79), PI + MF (9/79), and mixed types with ID component (22/79) [ID + PI (19/79), ID + MF (2/79), and ID + PI + MF (2/79)]. The trend of OS and survival rate of PI + MF was poor similar to that for PI or MF. PI + MF showed obviously shorter survival than ID (OS = 9 vs. 36 mo., *p* < 0.001), and mixed types with ID components (OS = 9 vs. 28 mo., *p* = 0.048); however, there was no significant difference when compared to PI (OS = 9 vs. 12, *p* = 0.066) or MF (OS = 9 vs. 12, *p* = 0.297) (Table 4; Figure 3). Multivariate analysis, thus, established that the new growth pattern subclassification was an independent factor for poor prognosis in dCCA (Supplementary Table 1).

**Table 4:** Overall survival of distal cholangiocarcinoma patients based on five subclassifications of growth pattern.

**Feature**	***N* (%) 79 (100)**	**OS (month)**	**Survival rate (years)**			**Univariate analysis HR (95%CI)**	***P***
			**1**	**3**	**5**		
Growth pattern (subgroup)							
ID	13 (16.5)	40	100%	61.5%	30.8%	1	
PI	25 (31.6)	8	28%	0%	0%	16.11 (6.60–39.34)	<0.001
MF	9 (11.4)	11	44.4%	0%	0%	11.43 (4.03–32.46)	<0.001
Mixed type without ID component (PI + MF)	9 (11.4)	9	30%	0%	0%	16.35 (5.86–45.64)	<0.001
Mixed types with ID component (ID + PI, ID + MF, ID + PI + MF)	23 (29.1)	27	95.2%	28.6%	4.8%	2.24 (1.01–4.96)	0.048

### Multivariate Analysis of Growth Pattern Subclassification and Significant Factor in Univariate Analysis

Growth patterns without ID showed high hazard ratios when compared to pure ID and ID components (Table 4; Figure 3). Patients were divided in 3 groups comprising pure ID (*n* = 13), with ID components (ID + PI, ID + MF, ID + PI + MF) (*n* = 23), and without ID components (PI, MF, PI + MF) (*n* = 43) (Supplementary Figure 1). Multivariate analysis was performed using features in Table 2, including surgical margin and TNM categories. Interestingly, results showed that growth patterns without ID components had higher HR ratio and significant statistical difference when compared to the reference group (ID) (HR = 12.14, *p* < 0.001); and that those with ID components showed significant difference and HR ratio higher than pure ID (HR = 2.49, *p* < 0.034) (Table 5). Therefore, we considered that the growth pattern could be useful for further subclassification of dCCA patients.

**Table 5:** Overall survival of distal cholangiocarcinoma patients based on five subclassifications of growth pattern and multivariate analysis.

**Feature**	***N* (79)**	**OS (months)**	**Multivariate analysis HR (95%CI)**	***P***
Surgical margin (R)				
R0	54	23	1	
R1	25	11	1.54 (0.89–2.66)	0.121
Growth pattern				
ID	13	40	1	
With ID components
(ID + PI, ID + MF,	23	27	2.49 (1.07–5.77)	0.034
ID + PI + MF)
Without ID components
(PI, MF, PI + MF)	43	9	12.14 (4.79–30.73)	<0.001
8th AJCC staging system				
T categories				
T1	10	31	1	
T2	38	14	1.54 (0.68–3.50)	0.301
T3	29	17	1.48 (0.63–3.49)	0.375
T4	2	–	–	–
Lymph node metastasis (N)				
N0	51	23	1	
N1	26	12	1.10 (0.63–1.92)	0.742
N2	2	–	–	–
Metastasis (M)				
M0	69	22	1	
M1	10	3	1.04 (0.47–2.33)	0.921

**Figure 4:**
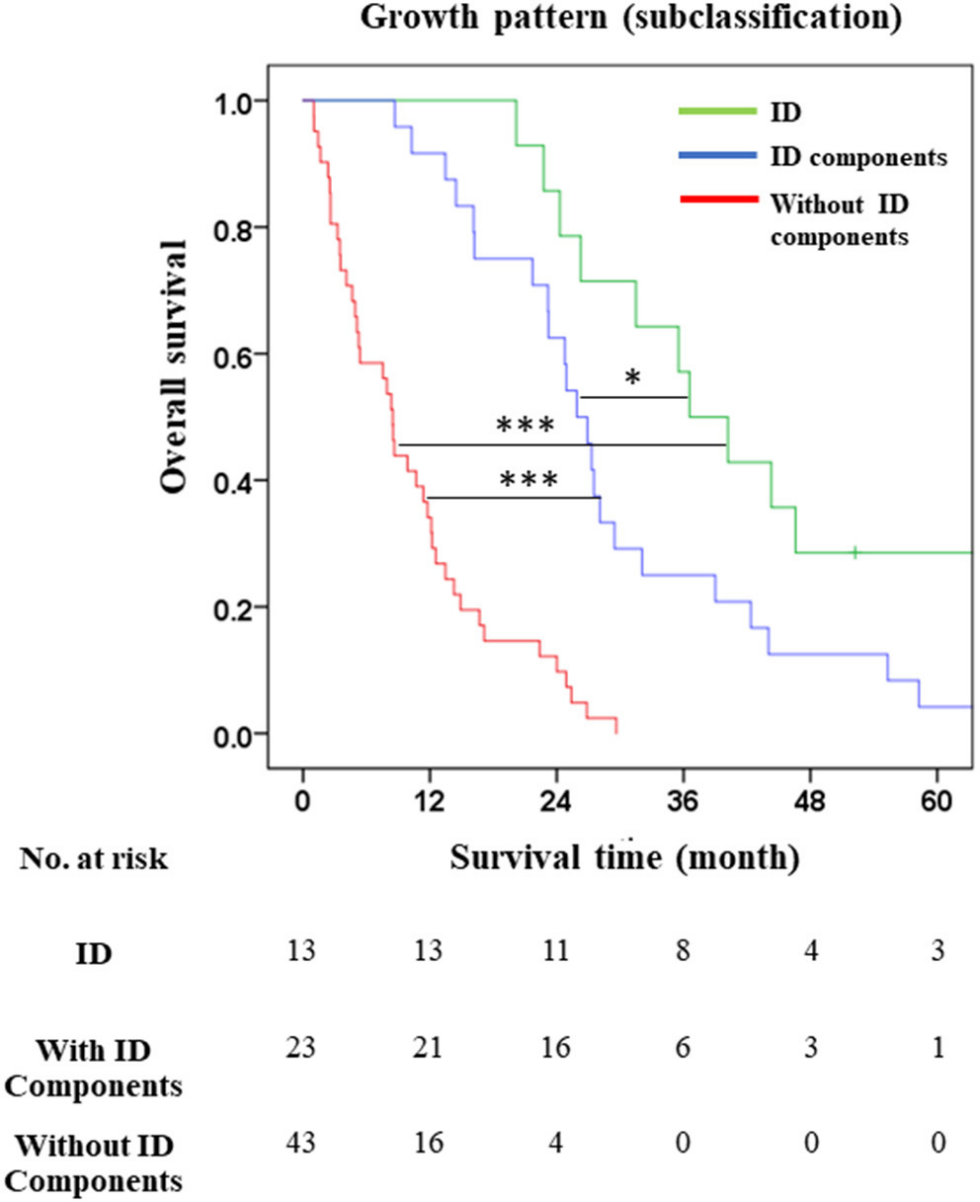
Overall survival of growth pattern subclassification in distal cholangiocarcinoma. *p < 0.05 and ***p < 0.001.

The growth pattern was separated into three groups: pure ID, with ID components, and without ID components. The survival analysis showed that the stratification performance based on subclassification of dCCA by growth pattern afforded good separation in each group with OS and survival rate trending downwards with the disappearance of ID component. The results showed that OS, HR and survival rate in pure ID were better than mixed types with ID components (OS = 40 vs. 27 mo., HR = 2.39, *p* = 0.030; and 1y = 100 vs. 92.2, 3y = 61.5 vs. 28.6, and 5y = 30.8 vs. 4.8%), and mixed types without ID components (OS = 40 vs. 9 mo., HR = 13.71, *p* < 0.001; and 1y = 100 vs. 38.2, 3y = 61.5 vs. 0%, and 5y = 30.8 vs. 0%) (Table 6). Moreover, we found that OS and survival rate of those with ID components were better than those without ID components (OS = 27 vs. 9 mo., *p* < 0.001). This finding showed that growth pattern subclassification was able to separate each group—ID vs. ID components (*p* = 0.048), ID vs. without ID components (*p* < 0.001), and ID components vs. without ID components (*p* < 0.001) (Figure 4).

**Table 6:** Overall survival and 1-, 3- and 5-year survival rates of distal cholangiocarcinoma patients with subclassification by growth pattern.

**Growth pattern classification**	***N* (%) 79 (100)**	**OS (month)**	**Survival rate**			**Univariate analysis HR (95%CI)**	***P***
			**1**	**3**	**5**		
ID	13 (16.5)	40	100%	61.5%	30.8%	1	
With ID components	23 (29.1)	27	92.2%	28.6%	4.8%	2.39 (1.09–5.24)	0.030
Without ID components	43 (54.4)	9	38.2	0%	0%	13.71 (5.87–32.01)	<0.001

*With ID components, ID + PI, ID + MF and ID + PI + MF; Without ID components, PI, MF and PI + MF*.

### Correlation of Growth Pattern With Clinicopathological Features in Distal Cholangiocarcinoma

The important question is whether there is any correlation between clinicopathological features indicating aggressive behavior and the groups of growth patterns signifying good and poor outcomes. Age, gender, tumor size, surgical margin, histological type, histological grade, T categories, lymph node metastasis, and distant metastasis were analyzed in conjunction with pure ID, with ID components (ID + PI, ID + MF, ID + PI + MF), and without ID components (PI, MF, PI + MF). Interestingly, the results revealed that those without ID components were significantly correlated with positive lymph node metastasis, N1, while pure ID and ID components were correlated with negative lymph node metastasis, N0 (*p* = 0.007) (Table 7). Moreover, results further showed that growth patterns without ID components correlated with lymph node and distant metastases, which are indicators of poor prognosis (Supplementary Table 2).

**Table 7:** Correlation between subclassification of growth patterns and clinicopathological features in distal cholangiocarcinoma.

**Feature**	**Subclassification of growth pattern**				***P***
	**ID *N* (%)**	+ ID^*^N (%)	−ID^**^N (%)	**Total *N* (%)**	
Age					
≤59	7 (53.8)	9 (39.1)	22 (51.2)	38 (48.1)	0.584
>59	6 (46.2)	14 (60.9)	21 (48.8)	41 (51.9)	
Gender					
Male	9 (69.2)	15 (65.2)	31 (72.1)	55 (69.6)	0.845
Female	4 (30.8)	8 (34.8)	12 (27.9)	24 (30.4)	
Tumor size^***^					
≤2	7 (87.5)	7 (58.3)	12 (50)	26 (59.1)	0.174
>2	1 (12.5)	5 (41.7)	12 (50)	18 (40.9)	
Surgical margin					
R0	11 (84.6)	18 (78.3)	25 (58.1)	54 (68.4)	0.095
R1	2 (15.4)	5 (21.7)	18 (41.9)	25 (31.6)	
Histological type					0.611
Papillary adenocarcinoma	5 (38.5)	7 (30.4)	17 (39.5)	29 (36.7)	
Tubular adenocarcinoma	5 (38.5)	11 (47.8)	16 (37.2)	32 (40.5)	
Papillotubular adenocarcinoma	2 (15.3)	4 (17.4)	3 (7)	9 (11.4)	
Adenocarcinoma, NOS	1 (7.7)	1 (4.3)	7 (16.3)	9 (11.4)	
Histological grade					
Welldifferentiated	12 (92.3)	21 (91.3)	37 (86)	70 (88.6)	0.733
Moderately/poorly differentiated	1 (7.7)	2 (8.7)	6 (14)	9 (11.4)	
8th AJCC					
T categories					
T1	3 (23.1)	4 (17.4)	3 (7)	10 (12.7)	0.310
T2	7 (53.8)	9 (39.1)	22 (51.2)	38 (48.1)	
T3	2 (15.4)	10 (43.5)	17 (39.5)	29 (36.7)	
T4	1 (7.7)	1 (2.3)	–	2 (2.5)	
N category					
N0	11 (84.6)	19 (82.6)	21 (48.8)	51 (64.6)	0.007
N1	2 (15.4)	4 (17.4)	22 (51.2)	28 (35.4)	
M category					
M0	13 (100)	22 (95.7)	13 (79.1)	69 (87.3)	0.05
M1	0 (0)	1 (4.3)	21 (20.9)	10 (12.7)	

*, *ID components*.

**, *Without ID components*.

***, *Unknown tumor size was not determined*.

## Discussion

In Southeast Asia, dCCA constitutes a minority of all CCAs and the incidence is decreasing in contrast to intrahepatic CCA. However, the clinical course of both forms of CCAs are equally dismal (16, 23). From 2002 to 2017, dCCA constituted 8% of CCA in this study cohort (15, 16). The OS and 5-year survival rate were very poor; OS was 17 mo., while 1, 3- and 5-year survival rates were 62%, 16.5% and 6.3%, respectively, after curative-intended resection (Supplementary Figures 2A,B) (18–20, 23).

Therefore, precise diagnosis and optimum treatment strategies are required for managing dCCA patients after resection. Pathological staging by AJCC/UICC staging system is essential for managing patients after surgery because it can predict the progression of the disease by clustering the patients leading to precise prognosis and treatment. Nowadays, the 8th edition AJCC staging system is used to stratify cancer patients worldwide for the treatment plan. The 8th edition was improved from the 7th AJCC staging system by changing T and *n* categories—incorporating depth of invasion in T1-T3 and number of lymph node metastasis (17, 41, 42). This enabled better separation of each stage in early (T1-T3) and late stages (N1-N2) of dCCA. However, there are increasing validation studies calling for either modification or additional factors to further improve the stratification ability of the 8th AJCC staging system. Min et al. demonstrated that the depth of invasion (DOI) values of 8th AJCC staging system for separating T1 (DOI <5 mm), T2 (DOI = 5–12 mm), and T3 (DOI > 12 mm) showed no significant difference in survival analysis in their cohort. They proposed a new T1-3 category based on new DOI; T1 (DOI <3 mm), T2 (DOI = 3–10 mm), and T3 (DOI > 10 mm) showed a significant correlation with survival rates (43). There is a suggestion from Park et al. that although DOI is useful to separate T1-3, the measurement of DOI does not need to be rigorously and stringently performed (31). Wu et al. in a study of 758 patients with dCCA to examine the optimal numbers of positive lymph nodes revealed that N1 with 1–2 node-positive (1–3 for 8th AJCC) and N2 ≥ 3 nodes positive (≥4 for 8th AJCC) can significantly separate survival time of N1 and N2 better than 8th AJCC staging system (29). Other studies propose additional factors, such as a prognostic factor, to improve prognosis in dCCA. Ji et al. found that neutrophil-to-lymphocyte ratio (NLR) was associated with poor prognosis (33). Furthermore, the cancer biomarker, serum carbohydrate antigen 19–9, used as a prognostic factor for poor survival of dCCA patients correlated well with regional lymph node metastases (34). Similarly, our study showed that 8th AJCC staging system was not able to classify T1-T3 (N0) *via* DOI, and it cannot significantly separate each T stage [(T1 vs. T2, OS = 32 vs. 20 mo., *p* = 0.055), (T1 vs. T3, OS = 32 vs. 24, *p* = 0.098) and (T2 vs. T3, OS = 20 vs. 24, *p* = 0.896)] (Supplementary Figure 3). N2 was found in a small number of patients (*n* = 2), and only N0 and N1 were available for classification. TNM staging by 8th AJCC showed poor stratification of dCCA patients, with only main stages I (9/79), II (57/79), and IV (10/79) available for calculation of survival time while III had too few patients (3/79). Our study showed that growth pattern, especially PI (25/79) and MF (9/79), was independent factor for poor survival of dCCA. PI + MF (9/79) when compared with ID, Patients with growth patterns comprising pure ID (13/79) or ID components (23/79) (ID + PI, ID + MF, ID + PI + MF) have better survival than PI, MF, and PI + MF (1, 44–46). Although for dCCA, PI type is the most common macroscopic growth pattern appearance (47), Tawarungruang et al. also found MF in their study of dCCA (48). They studied the influence of growth patterns in the three subtypes of CCAs, namely, iCCA, pCCA and dCCA, and found that ID in dCCA correlated with better survival time than PI and MF, as also observed in iCCA and pCCA. Literature supports our finding to propose growth pattern as a prognostic factor in dCCA. Lymph node metastasis comprised two groups—N0 (node-negative, 61/79) and N1 (node-positive 1–3 nodes, 26/79), while N2 had too few cases (node-positive ≥ 4 nodes, 2/79). Therefore, the *n* category was not applied in this study for two reasons—firstly, N2 had too few cases, thus, precluding estimation of OS or TNM staging for stage III; secondly, this study proposes growth pattern as a prognostic factor for separating the good and poor outcomes of dCCA patients.

The subclassification based on growth pattern divided dCCA patients into three groups comprising pure ID (*n* = 13), and mixed types with ID components (*n* = 23) (ID + PI, ID + MF, ID + PI + MF), and without ID components (*n* = 43) (PI, MF, PI + MF). This model was more suited to our cohort of dCCA patients than the 8th AJCC staging system (Figures 3, 4); separating patients into those with well (ID), median (ID components), and poor (without ID components) survival (Figure 4). Based mainly on ID composition, the dCCA patients were divided into two groups comprising those with ID components (*n* = 36) (pure ID, ID + PI, ID + MF, ID + PI + MF) who showed good survival time and rate, and those without ID components (*n* = 43) (PI, MF, PI + MF) who showed otherwise. This finding has been supported by previous reports on other subtypes of CCA (44, 48, 49). They showed that iCCA patients with PI, MF and PI + MF growth patterns fared poorly when compared with iCCA patients with ID. Notably, growth patterns comprising PI, MF, and PI + MF, showed significant correlation with positive lymph node and distant metastasis, while ID components showed correlation with negative lymph node and distant metastasis (Table 6; Supplementary Table 2). This information emphasizes that the growth pattern without ID components represents late-stage disease according to 8th AJCC staging system. In other words, ID components indicate early-stage disease with a better outcome for dCCA patients. This information is helpful for prognosticating the progression of disease and guiding treatment options.

From the perspective of this study, we believe that with validation from internal and external cohorts, this model can be put into practice at our institute. Since growth pattern is routinely recorded in pathological gross examination of CCA resection specimens, it could be benefitial for prognostication and planning for treatment or palliative care. There is a prospect for inclusion of growth pattern into the next revision of the AJCC staging system to improve stratification ability and provide potential treatment in CCA patients.

The limitation of this study is that it is a pilot study on a Thai cohort performed at a single institution in Northeast Thailand. The small number of dCCA cases leads to a lack of pathological data on T and *n* (N2) categories and TNM staging, which does not adequately represent the rest of the patient population. Moreover, subgroup analysis of growth pattern in clinical features could be not calculated due to small number of dCCA cases in some features. Therefore, this investigation will be firstly considered when we get more dCCA patient cases. Information on postoperative complications which highly impact prognosis was not available in our database. Additional factors which might affect outcomes, such as body mass index, smoking, HBV, HCV, liver function tests, and total cholesterol, are also not available. Therefore, validation of growth pattern subclassification in larger cohorts in Thailand and in other communities is necessary to evaluate stratification performance.

In summary, this study showed that growth pattern/s, particularly, PI, MF and PI + MF, is an independent factor for poor survival in dCCA; conversely, presence of ID component is a predictor for good prognosis. Moreover, the subclassification based on growth pattern was able to separate dCCA patients into those with good (ID and ID components) and poor (without ID components) outcome. This study also found that growth patterns without ID components (PI, MF, PI + MF) correlated with lymph node and distant metastasis. Growth pattern is a potential candidate for inclusion into the current staging system; however, much more work and validation are required.

## Data Availability Statement

The original contributions presented in the study are included in the article/Supplementary Material, further inquiries can be directed to the corresponding author/s.

## Ethics Statement

This study was approved by the Ethics Committee for Human Research, Khon Kaen University (HE641499). The patients/participants provided their written informed consent to participate in this study.

## Author Contributions

PS, SK, CA, SS, and PI: conceptualization. SK: funding acquisition and supervision. PS, SK, CA, SS, PI, WK, WB, NK, AT, AJ, VT, TS, VL, KE, WL, and JP: sample collection and diagnosis. PS, SK, CA, and PP: analysis and interpretation of data. PS, SK, CA, SS, PI, and PP: writing original draft. PS, SK, CA, SS, PI, WK, WB, MT, and PP: writing review and editing. All authors approved the final version of the manuscript.

## Funding

This research was supported by the Basic Research Fund of Khon Kaen University under Cholangiocarcinoma Research Institute (CARI), Khon Kaen University, Khon Kaen, Thailand. Furthermore, this research was funded by Cholangiocarcinoma Screening and Care Program (CASCAP), Khon Kaen University, Khon Kaen, Thailand.

## Conflict of Interest

The authors declare that the research was conducted in the absence of any commercial or financial relationships that could be construed as a potential conflict of interest.

## Publisher's Note

All claims expressed in this article are solely those of the authors and do not necessarily represent those of their affiliated organizations, or those of the publisher, the editors and the reviewers. Any product that may be evaluated in this article, or claim that may be made by its manufacturer, is not guaranteed or endorsed by the publisher.
