# Supplementary material for: Prognostic Significance of Growth Pattern in Predicting Outcome of Opisthorchis viverrini-Associated Distal Cholangiocarcinoma in Thailand
Source: Front Public Health. 2022 May 16;10:816028. doi: 10.3389/fpubh.2022.816028 (PMC9149579; doi:10.3389/fpubh.2022.816028)
Supplement: Supplementary file 1 [file Data_Sheet_1.docx]

Supplementary Material

## Supplementary Figures


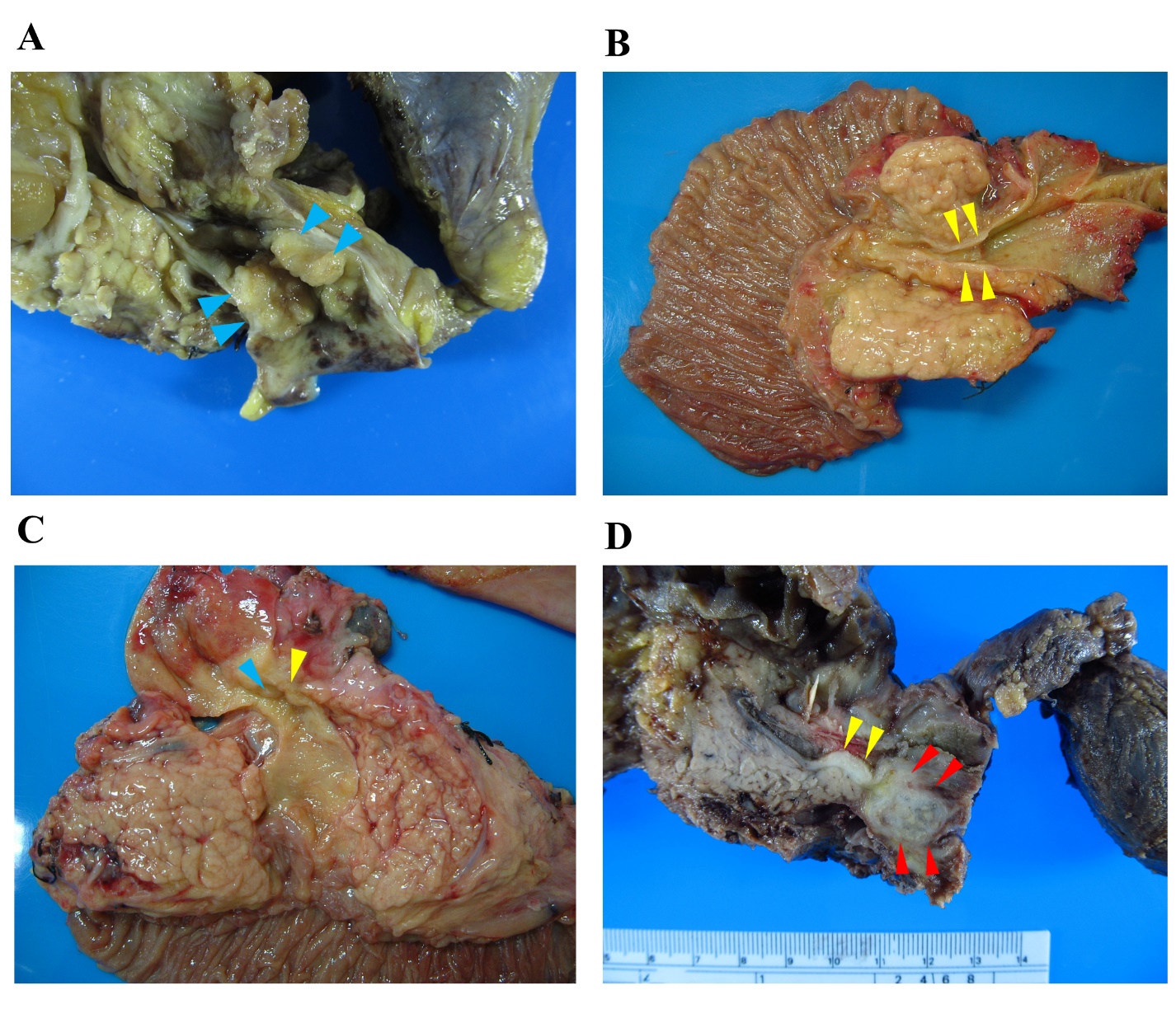


**Supplementary Figure 1.** Representative figures of resections for distal cholangiocarcinoma demonstrating the various growth patterns. (A) Intraductal (ID), (B) Periductal infiltrating (PI), (C) ID + PI, and (D) PI + Mass-forming (MF). Blue, yellow and red arrowheads represent ID, PI and MF, respectively.


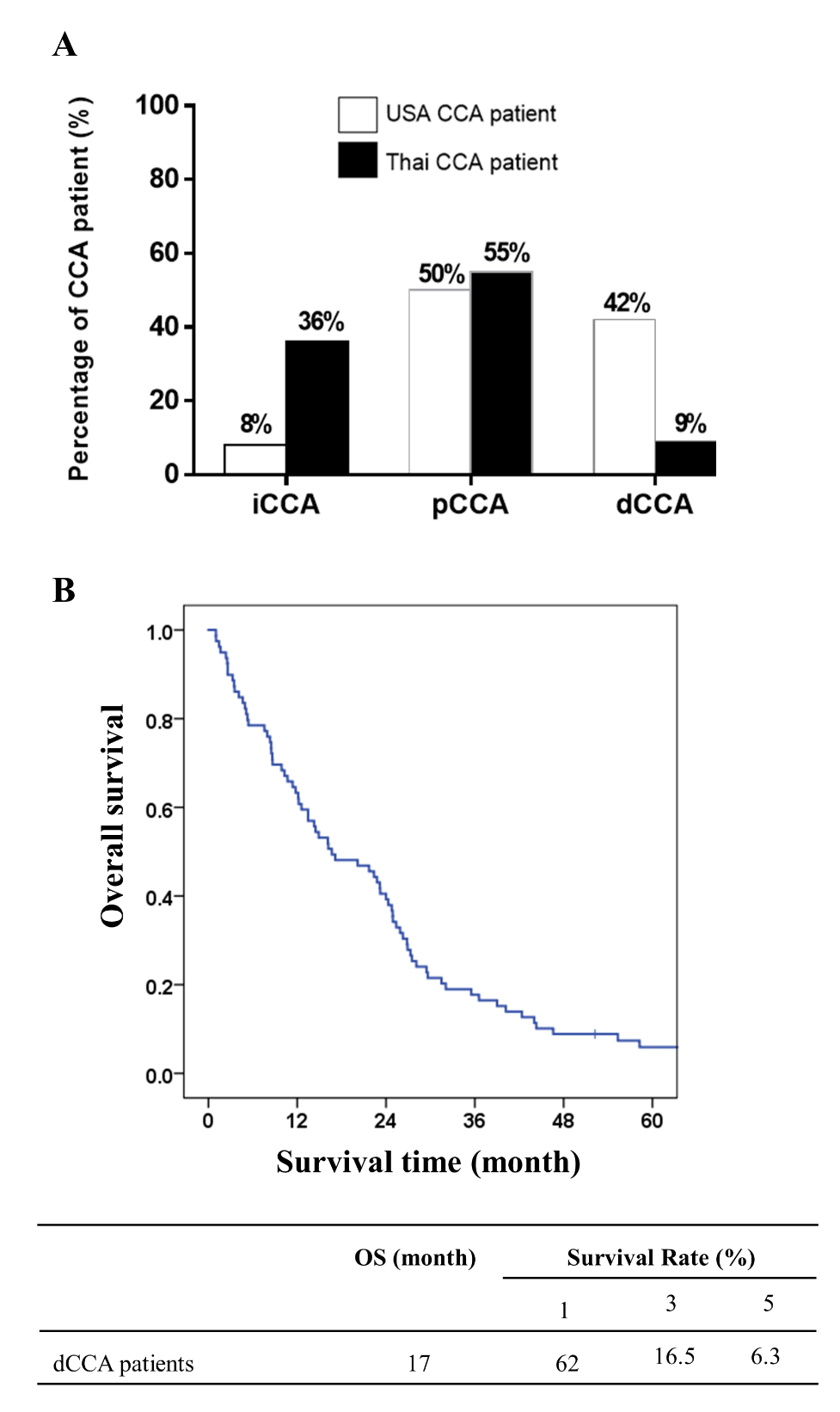


**Supplementary Figure 2.** (A) Comparison of incidence rate of distal cholangiocarcinoma between Thailand and USA. (B) Overall survival and 1-, 3- and 5-survival rate of distal cholangiocarcinoma patients.


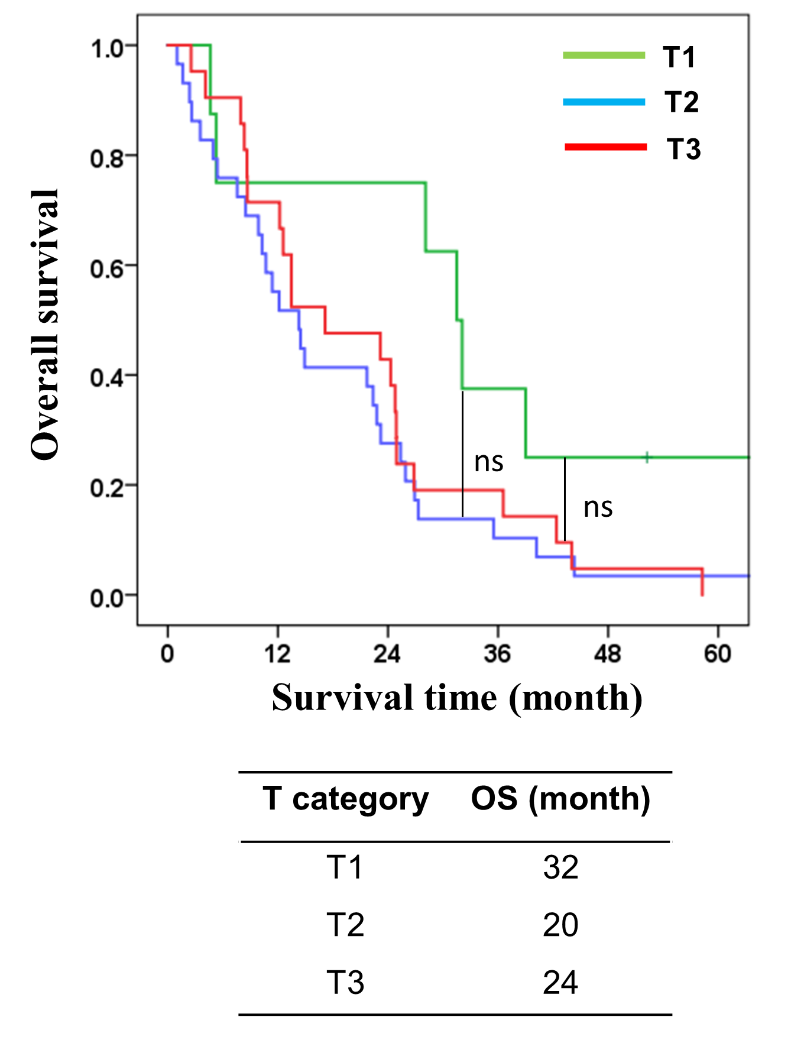


**Supplementary Figure 3.** Comparison of T1, T2 and T3 categories with N0 of distal cholangiocarcinoma patients.

## Supplementary Tables

**Supplemental table 1.** Multivariate analysis of subgroups of growth pattern and significant features from univariate analysis in distal cholangiocarcinoma patients.

| **Features** | **HR (95%CI)** | ***P*** |
| --- | --- | --- |
| Surgical margin |  |  |
| R0 | 1 |  |
| R1 | 1.549 (0.885-2.713) | 0.126 |
| Growth pattern |  |  |
| ID | 1 |  |
| PI | 16.244(5.932-44.479) | **<0.001** |
| MF | 9.139 (3.073-27.181) | **<0.001** |
| PI+MF | 15.16 (4.692- 48.982 ) | **<0.001** |
| Mixed type with ID components | 2.366 ( 1.004-5.577) | **<0.05** |
| T category |  |  |
| T1 | 1 | 0.814 |
| T2 | 1.5 (0.657-3.428) | 0.336 |
| T3 | 1.424 (0.597-3.396) | 0.425 |
| T4 | - | - |
| N category |  |  |
| N0 | 1 |  |
| N1 | 1.107 (0.617-1.984) | 0.734 |
| M category |  |  |
| M0 | 1 |  |
| M1 | 0.864(0.367-2.035) | 0.738 |

**Supplemental table 2.** The correlation between growth pattern with lymph node and

distant metastasis in distal cholangiocarcinoma patients

| **Feature** | **Subclassification of growth pattern** | | | ***P*** |
| --- | --- | --- | --- | --- |
|  | **With ID**  **n (%)** | **Without ID**  **n (%)** | **Total**  **n (%)** |  |
| N category |  |  |  |  |
| N0 | 30  (83.3) | 21  (48.8) | 51  (64.6) | <0.01 |
| N1 | 6  (16.7) | 22  (51.2) | 28  (35.4) |  |
| M category |  |  |  |  |
| M0 | 35  (97.2) | 34  (79.1) | 69  (87.3) | <0.05 |
| M1 | 1  (2.8) | 9  (20.9) | 10  (12.7) |  |
